# Electronic health record-based identification of inpatients receiving antibiotic treatment for community-acquired pneumonia

**DOI:** 10.1017/ash.2024.41

**Published:** 2024-04-19

**Authors:** David Yang, Leigh Cressman, Keith W. Hamilton, Lauren Dutcher

**Affiliations:** 1 University of Pennsylvania Perelman School of Medicine, Philadelphia, PA, USA; 2 Department of Biostatistics, Epidemiology, and Informatics, University of Pennsylvania Perelman School of Medicine, Philadelphia, PA, USA; 3 Division of Infectious Diseases, Department of Medicine, University of Pennsylvania Perelman School of Medicine, Philadelphia, PA, USA

## Abstract

We conducted a retrospective study to assess performance of provider-selected antibiotic indication (PSI) in identifying hospitalized adults with community-acquired pneumonia. PSI showed moderate sensitivity (64.4%) and high specificity (96.3%). PSI has potential utility for targeted real-time antibiotic stewardship interventions, though future research should investigate methods to improve sensitivity.

## Introduction

Community-acquired pneumonia (CAP) is one of the most common indications for antibiotic prescribing in the inpatient setting.^1^ However, inappropriate antibiotic prescribing for CAP is common, particularly excess duration.^2^ Antibiotic stewardship (AS) programs reduce guideline-discordant antibiotic prescribing and improve outcomes for patients with CAP.^3^ Quickly identifying patients hospitalized with CAP in the electronic health record (EHR) who could benefit from stewardship remains challenging.^4^ Prior studies have used ICD-10 codes and claims data to identify pneumonia cases, which show mixed performance and cannot identify encounters at the point of care.^5,6^ An alternative approach that has not been well-validated is using a provider-selected indication (PSI) for antibiotic orders. This would allow for real-time identification of patients treated for CAP who can be assessed for AS interventions. Our study aims to assess the ability of PSI for antibiotic orders to identify CAP cases.

## Methods

### Study design and population

In our health system, prescribers have been required to select an indication for antibiotic orders from a pre-specified list since 2017 (Supplement Table 1). While some indications are included in order sets, there is no pneumonia-specific order set. To assess the performance of PSI in identifying patients receiving treatment for CAP, we conducted a retrospective validation study of patients ≥18 years old admitted to one of three acute care hospitals within an academic health system from January 1, 2019 through December 31, 2021. Patients were eligible if they received at least one systemic antibiotic within 48 hours of admission; antibiotics never used to treat CAP were excluded (Supplement Table 2). We chose this source population to reflect a broad population from which AS teams might be seeking to identify pneumonia patients. Encounters with insufficient documentation of intended antibiotic use were excluded. A random sample was selected with a target of 440 encounters.

### Definitions

PSI for community-acquired lower respiratory tract infection (CA-LRTI) was identified if at least one antibiotic administered in the first 48 hours of admission had a selected indication of “Pneumonia/lower respiratory infection–community acquired.” The only other lower respiratory tract option was “Pneumonia/lower respiratory infection–healthcare associated.” The reference standard was defined as documented intention to treat for pneumonia by inpatient provider(s) within 48 hours of admission, as assessed by manual review of EHR notes; accuracy of the CAP diagnosis was not assessed. Any documented terminology for pneumonia except hospital-acquired pneumonia (HAP) or ventilator-associated pneumonia (VAP) was counted in this reference standard.

### Data collection

Demographic and antibiotic administration data were extracted from the EHR (Epic Clarity). Data from chart abstraction were stored in REDCap hosted at the University of Pennsylvania and included intended antibiotic indication(s) and chest radiology report results.^7^ A medical student (DY) was trained by two infectious disease physicians (KH and LD) to conduct chart abstraction. Ten charts were reviewed by both LD and DY for training, and ambiguous cases were reviewed by KH or LD.

### Statistical analysis

PSI CA-LRTI was compared against the reference standard by calculating sensitivity, specificity, and positive and negative predictive values. Secondary analyses included: (1) Including encounters documenting the terms HAP and VAP in the reference standard to determine the effect on sensitivity; (2) Adjusting PSI to include healthcare-associated LRTI (PSI HA-LRTI) to assess provider differentiation among CA-LRTI and HA-LRTI; and (3) Assessing performance characteristics of pneumonia ICD-10 codes using the same reference standard for comparison (Supplement Table 3). The proportion of patients with a new/increased infiltrate on chest radiograph (*x*-ray) or computed tomography scan in the first 48 hours was also assessed.

## Results

There were 442 encounters screened with 440 included and two excluded due to insufficient documentation. Demographic characteristics and comorbidities are described in Table 1. Seventy-three (16.5%) patients were treated for lower respiratory tract infection (LRTI). Sixty-four (87.7%) of LRTI-treated patients were treated for pneumonia, 10 (13.7%) for COPD exacerbation, 1 (1.4%) for bronchiectasis flare, and 2 (2.7%) for other conditions. The most common antibiotics used for LRTI were vancomycin (25.0%), 4th generation cephalosporins (24.4%), macrolides (16.5%), and 3rd generation cephalosporins (12.8%).

**Table 1.** Demographics and clinical characteristics of included patients

Demographics	n = 440
Age, years, median (IQR)	61 (44.0–71.3)
Sex, n (%)	
Male	208 (47.3)
Female	232 (52.7)
Race, n (%)	
White	229 (52.0)
Black	170 (38.6)
Asian	12 (2.7)
East Indian	1 (0.2)
Other	8 (1.8)
Unknown	20 (4.5)
Ethnicity, n (%)	
Non-Hispanic non-Latino	413 (93.9)
Hispanic or Latino	18 (4.1)
Unknown	9 (2.0)
Hospital length of stay, days, median (IQR)	4.2 (2.7–8.1)
Comorbidities, n (%)	
Diabetes	135 (30.7)
Cirrhosis	12 (2.7)
Cystic fibrosis	2 (0.5)
COPD/bronchiectasis	56 (12.7)
Solid organ transplant	24 (5.5)
Bone marrow transplant	9 (2.1)
HIV	10 (2.3)
Chronic kidney disease	86 (19.6)
Malignancy	100 (22.7)
Congestive heart failure	72 (16.4)
Antibiotics used per patient, median^a^ (IQR)	1 (1–2)
Antibiotic classes used frequency^a^ (% of total antibiotics prescribed)	
Glycopeptide	164 (21.1)
1^st^ and 2^nd^ generation cephalosporin	128 (16.4)
3^rd^ generation cephalosporin	77 (9.9)
4^th^ generation cephalosporin	109 (14.0)
Penicillin + beta lactamase inhibitor (non-anti-pseudomonal)	34 (4.4)
Penicillin + beta lactamase inhibitor (anti-pseudomonal)	35 (4.5)
Nitroimidazole	47 (6.0)
Macrolide	46 (5.9)
Penicillins	35 (4.5)
Fluoroquinolone	27 (3.5)
Other	77 (9.9)
Total	779 (100)

a Includes antibiotic use for all included patients, not just patients with pneumonia.

PSI CA-LRTI had a sensitivity of 64.4% (95% CI, 59.9–68.9) and a specificity of 96.3% (95% CI, 94.6–98.1) for the detection of CAP treatment, with positive and negative predictive values reported in Table 2. Of 21 false negatives, all except one had HA-LRTI or sepsis as a selected indication for at least one antibiotic order. PSI CA-LRTI had comparable performance to pneumonia ICD-10 codes (Table 2). Adjusting the PSI to include selection of either CA or HA-LRTI increased sensitivity to 86.4% (95% CI, 83.2–89.6) but decreased specificity to 92.9% (95% CI, 90.5–95.3). Including all pneumonia terminology in the reference standard did not significantly change results (Table 2). The overall accuracy of PSI (sum of true positives and true negatives divided by total included) was 92.0%.

**Table 2.** Performance measures of methods for identifying community-acquired pneumonia

Test	Reference standard	Sensitivity (%) (95% CI)	Specificity (%) (95% CI)	PPV (%) (95% CI)	NPV (%) (95% CI)
Provider-selected antibiotic indication for CA-LRTI	Provider intention to treat pneumonia with HAP/VAP terminology excluded	64.4 (59.9–68.9)	96.3 (94.6–98.1)	73.1 (68.9–77.2)	94.6 (92.5–96.7)
Provider-selected antibiotic indication for CA-LRTI	Provider intention to treat pneumonia (any terminology)	64.1 (59.6–68.6)	97.1 (95.5–98.7)	78.9 (75.0–82.7)	94.1 (91.9–96.3)
Provider-selected antibiotic indication for CA-LRTI or HA-LRTI	Provider intention to treat pneumonia with HAP/VAP terminology excluded	86.4% (83.2–89.6)	92.9% (90.5–95.3)	65.4% (60.9–69.8)	97.8% (96.4–99.1)
Prescence of pneumonia ICD-10 code	Provider intention to treat pneumonia with HAP/VAP terminology excluded	61.0 (56.5–65.6)	95.3 (93.3–97.3)	66.7 (62.3–71.1)	94.0 (91.8–96.3)

Note. CA-LRTI, community-acquired lower respiratory infection; HA-LRTI, healthcare-associated lower respiratory infection; VAP, ventilator-associated pneumonia; PPV, positive predictive value; NPV, negative predictive value.

We found that 86.2% of patients whom the provider treated for CAP had new or increased infiltrates noted on a radiology report. The EHR-based methods of CAP identification showed a similar proportion of new infiltrates, though including HA-LRTI lowered the proportion (Supplement Figure 1). Frequencies of terminology used in provider documentation for pneumonia are provided in Supplement Figure 2.

## Discussion

In this study, we showed that PSI of CA-LRTI had moderate sensitivity and high specificity for identifying CAP-treated cases; given the relatively low prevalence of CAP in the population, this translated to a moderate PPV and fairly high NPV.

We found that sensitivity was primarily lowered because of (1) use of sepsis as an antibiotic indication early during admission and (2) lack of differentiation between HAP and CAP when selecting the indication, which was supported by an increase in sensitivity when using both CA and HA-LRTI PSIs. Decreases in PSI accuracy for similar reasons have been reported by other studies.^8,9^ The Infectious Disease Society of America removed the term “healthcare-associated pneumonia” from guidelines in 2016.^10^ While this was not updated in our EHR antibiotic indications during the study period, it has since occurred to improve accurate categorization of pneumonia.

Like other studies assessing PSI accuracy, we show a high agreement rate (92%) between PSI and clinical documentation.^8,9^ Our study additionally calculates the sensitivity and specificity of PSI, which better assesses PSI performance for potential stewardship activities within a general population of patients receiving antibiotics. Though the PPV was only moderate, PSI could likely be used to increase the efficiency of identifying pneumonia cases for stewardship purposes, acknowledging that some non-pneumonia cases would still be identified. Additionally, despite the high NPV, not all pneumonia cases will be identified, and supplementation with other identification methods would be warranted. While PSI has similar sensitivity to ICD-10 codes for identifying CAP, PSI allows real-time identification of patients treated for CAP.

Our study has several limitations. Since the provider’s intention to treat CAP was the reference standard rather than an objective definition, some included patients may not in fact have pneumonia. Most providers used the general term “pneumonia” rather than “community acquired pneumonia,” which could result in misclassification of some encounters. Additionally, our antibiotic indication field may not be generalizable to other institutions.

In summary, we show PSI for antibiotic orders has potential for usage in real-time AS intervention for CAP patients. Methods with higher sensitivity should continue to be explored.

## Supporting information

Yang et al. supplementary material Yang et al. supplementary material

## Supplementary material

For supplementary material accompanying this paper visit http://doi.org/10.1017/ash.2024.41. click here to view supplementary material
