# Supplementary material for: Electronic health record-based identification of inpatients receiving antibiotic treatment for community-acquired pneumonia
Source: Antimicrob Steward Healthc Epidemiol. 2024 Apr 19;4(1):e53. doi: 10.1017/ash.2024.41 (PMC11036418; doi:10.1017/ash.2024.41)
Supplement: Yang et al. supplementary material [file S2732494X2400041Xsup001.docx]

**Supplement Table 1.** Provider-selected antibiotic indications during study period

| **Provider Selected Antibiotic Indications** |
| --- |
| Pneumonia/Lower respiratory infection   1. Community-acquired (onset < 72 hours after admission, no recent healthcare exposure) 2. Healthcare-associated (onset = 72 hours after admission or recent healthcare exposure) |
| Abdominal/pelvic   1. Community-acquired (onset < 72 hours after admission, no recent healthcare exposure) 2. Healthcare-associated (onset = 72 hours after admission or recent healthcare exposure) |
| Bloodstream infection |
| Bone/joint infection |
| Central nervous system |
| Endocarditis |
| ENT/OMFS/Ophtho |
| Genital infection |
| Gastrointestinal |
| Neutropenic fever |
| Other (specify below)   - Free text |
| Prophylaxis for immunosuppression |
| Severe sepsis/septic shock |
| Skin and soft tissue infection |
| Upper respiratory tract infection |
| Prophylaxis for surgery |
| Urinary tract infection   1. Uncomplicated cystitis, community-acquired (onset <72 hours after admission, no recent healthcare exposure) 2. Complicated cystitis, community-acquired (onset <72 hours after admission, no recent healthcare exposure) 3. Pyelonephritis, community-acquired (onset <72 hours after admission, no recent healthcare exposure) 4. Healthcare-associated cystitis or pyelonephritis |
| Unknown source |

**Supplement Table 2.** List of systemic antibiotics that were not counted when defining the inclusion criteria. All other systemic (oral, enteral, intravenous, and intramuscular routes) were included in the list for the inclusion criteria.

| **Systemic Antibiotics Excluded from Inclusion Criteria** |
| --- |
| Daptomycin  Erythromycin  Ethambutol  Fidaxomicin  Fosfomycin  Isoniazid  Nitrofurantoin  Pyrazinamide  Rifabutin  Rifampin  Rifapentine  Rifaximin  Vancomycin oral/enteral (not IV) |

**Supplement Table 3.** ICD-10 codes for pneumonia

| **ICD-10 Code for Pneumonia** |
| --- |
| J09.X1, J10.00, J10.01, J10.08, J11.00, J11.08, J12.0, J12.1, J12.2, J12.3, J12.8, J12.81, J12.82, J12.89, J12.9, J13, J14, J15, J15.1, J15.2, J15.21, J15.211, J15.212, J15.29, J15.3, J15.4, J15.5, J15.6, J15.7, J15.8, J15.9, J16.8, J16.0, J17, J18.0, J18.1, J18.8, J18.9, A37.01, A37.11, A37.81, A37.91, A48.1, J22 |

**Supplement Figure 1.** Proportion of patients with infiltrates on chest imaging by method of identification of community acquired pneumonia (CAP)


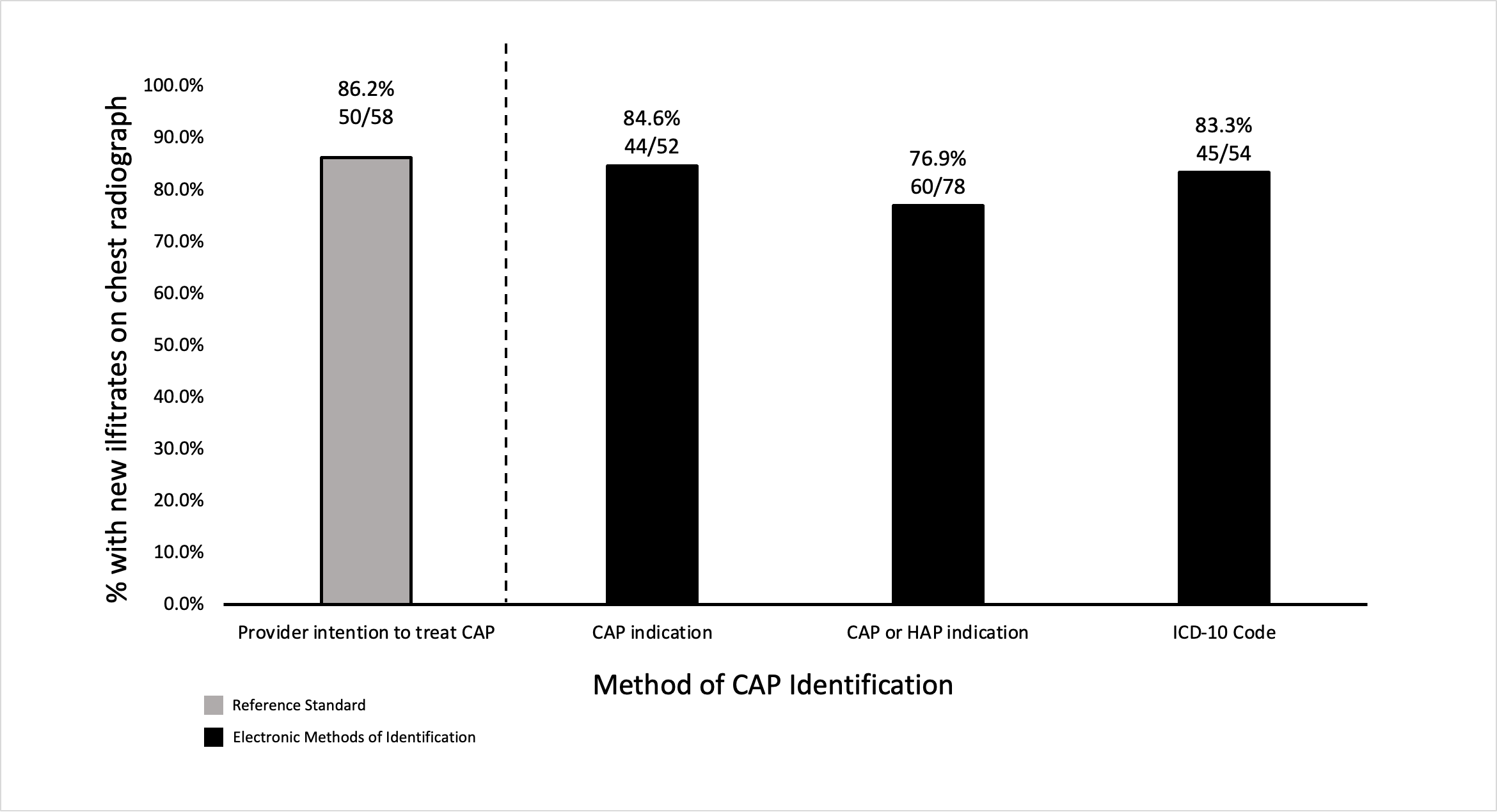


CAP, community-acquired pneumonia; HAP, healthcare-associated pneumonia

**Supplement Figure 2.** Distribution of pneumonia terminology among sixty-four patients with pneumonia by chart review


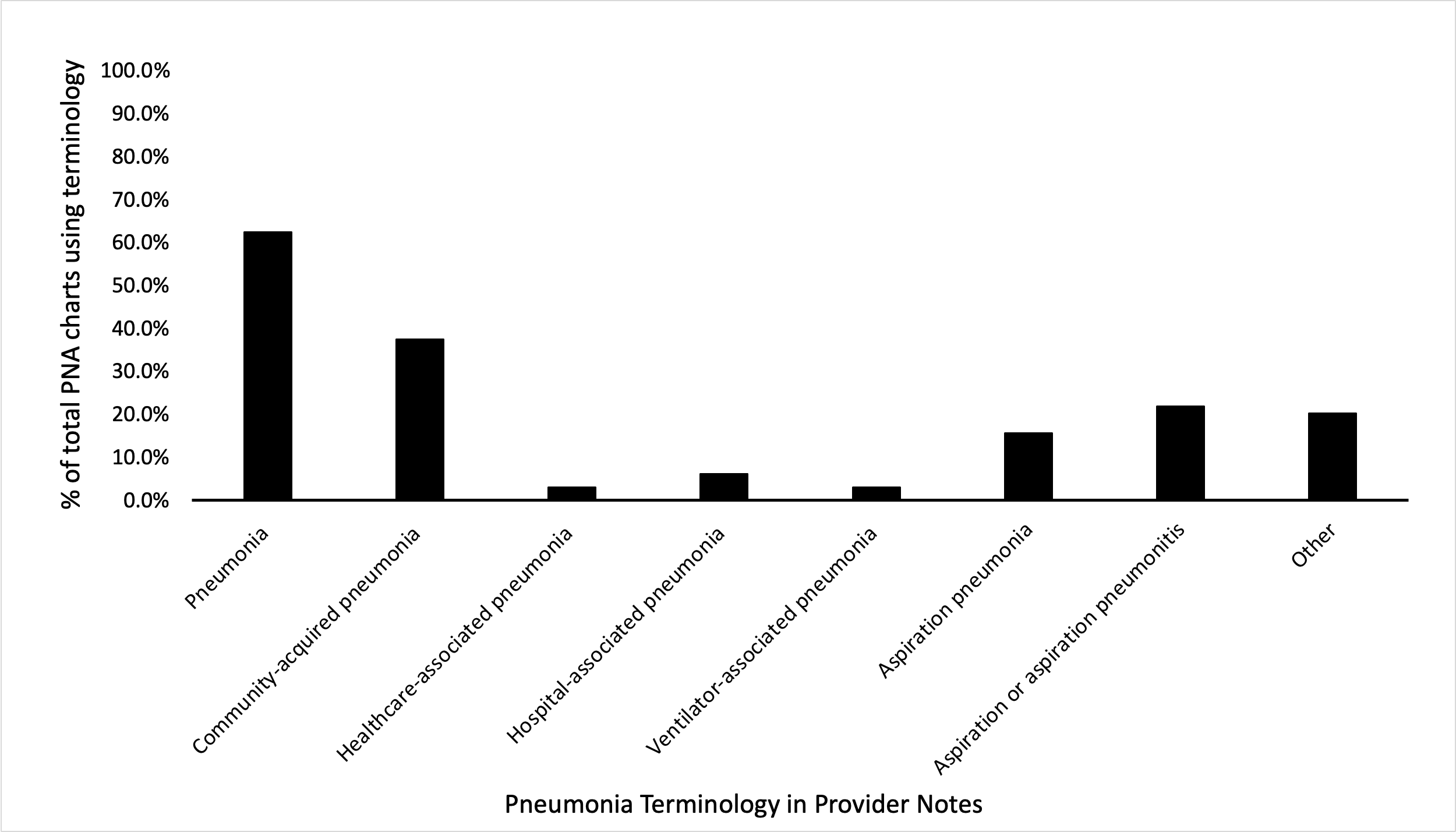


PNA, pneumonia

Note: the “other” category includes the following: COVID-19 pneumonia, 9 (64.3%); post-obstructive pneumonia, 2 (14.3%); *Pneumocystis jiroveci* pneumonia, 1 (7.1%); hemorrhagic pneumonia, 1 (7.1%); post-viral bacterial pneumonia, 1 (7.1%)

“

.
